# Radiofrequency ablation combined with toripalimab for recurrent hepatocellular carcinoma: A prospective controlled trial

**DOI:** 10.1002/cam4.6602

**Published:** 2023-10-10

**Authors:** Zhenyu Wen, Junxiao Wang, Bo Tu, Yane Liu, Yuqing Yang, Li Hou, Xiang Yang, Xiaoyan Liu, Hui Xie

**Affiliations:** ^1^ Department of Public Health Jilin University Jilin China; ^2^ Aerospace Medical Center Aerospace Center Hospital Beijing China; ^3^ Department of Infectious Diseases Fifth Medical Center of Chinese PLA General Hospital Beijing China; ^4^ Department of Oncology Fifth Medical Center of Chinese PLA General Hospital Beijing China; ^5^ Department of Hepatology Fifth Medical Center of Chinese PLA General Hospital Beijing China

**Keywords:** anti‐PD‐1, prospective study, radiofrequency ablation, recurrent hepatocellular carcinoma, toripalimab

## Abstract

**Objective:**

The effectiveness and security of radiofrequency ablation (RFA) in combination with toripalimab (anti‐PD‐1) for the treatment of recurrent hepatocellular carcinoma (HCC) was studied in this article.

**Methods:**

Total of 40 patients were enrolled in the study between September 2019 and November 2021. Data follow‐up ends in April 2022. The study’s main focus is on recurrence free survival (RFS), while the secondary objectives was safety. Chi‐square tests, Kaplan‐Meier, and Cox proportional hazards models were utilized to analyze the data.

**Results:**

The median follow‐up period was 21.40 months, and the median RFS was 15.40 months in the group that received combination therapy, which was statistically significantly different (HR: 0.44, *p* = 0.04) compared with the RFA group (8.2 months). RFS rates (RFSr) at 6, 12 and 18 months in the combination therapy groups and RFA groups were 80% vs 65%, 62.7% vs 35% and 48.7% vs 18.8%, respectively. Between the two groups, significant difference of RFSr was found at 18 months (*p* = 0.04). No statistical differences were observed between the two groups in terms of safeness (*p* > 0.05). The subgroup analysis indicated that the combination of RFA and anti‐PD‐1 led to better RFS than RFA alone. Moreover, patients benefited more from combination therapy in the groups younger than 60 years (HR: 0.26, *p* = 0.018), male (HR: 0.32, *p* = 0.028) and Child‐Pugh grade A (HR: 0.38, *p* = 0.032).

**Conclusions:**

Combining RFA with anti‐PD‐1 showed improved RFS and was deemed safe for patients with recurrent HCC who had previously undergone RFA treatment alone.

## Graphical abstract

Radiofrequency ablation (RFA) combined with toripalimab (anti‐PD‐1) is effective and safe in the treatment of recurrent hepatocellular carcinoma (HCC). Short‐term anti‐PD‐1 in combination with RFA may improve patient prognosis in patients with recurrent HCC.

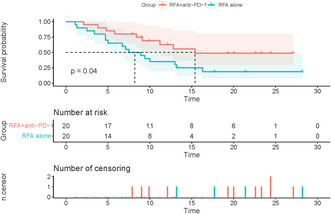

## 1 INTRODUCTION

In China, hepatocellular carcinoma (HCC) is a highly lethal and prevalent form of cancer with a mortality and morbidity rate that is among the highest in the world. Despite surgical resection being the preferred treatment for HCC, only a small percentage of patients (approximately 20%–30%) are able to undergo this procedure due to factors such as underlying hepatitis B infection, combined cirrhosis and liver insufficiency, multicentric tumor occurrence, and early tumor spread and metastasis, which are present in over 90% of cases.^1^, ^2^

Minimally invasive tumor treatment has developed remarkably during the past 20 years or so, and the guidelines for the treatment of HCC now include transcatheter arterial chemoembolization (TACE) and radiofrequency ablation (RFA).^3^ Currently, RFA has become the third major treatment for HCC next to surgical resection and interventional therapy, and it is considered as another definitive treatment for small HCC excluding surgery and liver transplantation because of its precise efficacy, especially in the treatment of small HCC, where the efficacy of RFA is similar to that of surgical resection.^4^, ^5^ Studies conducted both domestically and internationally have demonstrated that RFA is capable of killing HCC cells while also stimulating targeted anti‐tumor immune responses.^6^, ^7^, ^8^ Therefore, the anti‐tumor immune mechanism of RFA is becoming a new research hotspot in the field of HCC.^9^

Although RFA has the potential to boost the anti‐tumor immune response in HCC patients, typically the resulting immune response is insufficient to prevent tumor recurrence.^10^ After RFA, the 5‐year recurrence rate is still as high as 50%–80%,^11^, ^12^ due to the advantages of good reproducibility, less trauma and fewer complications of local ablation,^13^ and the option to continue local ablation therapy after recurrence is still a better choice.^14^ however, the recurrence risk still exists after another local ablation therapy.^15^ Therefore, in clinical practice, postoperative recurrence of RFA is still a challenge. The focus of clinical attention has been on how to optimize the treatment strategy to reduce recurrence after RFA.

Targeted immunotherapy against toripalimab (anti‐PD‐1) and its ligand PD‐L1 has demonstrated effective and lasting results in treating various types of tumors. The Food and Drug Administration has approved programmed death protein 1 (PD‐1) or PD‐L1 inhibitors for several indications.^16^, ^17^, ^18^, ^19^ Tumor immunotherapy has become another important tool in the field of tumor treatment after surgery, chemotherapy, radiotherapy, and targeted therapy.

Anti‐PD‐1 is a selective recombinant humanized monoclonal antibody that targets PD‐1. Which binds to PD‐1 and blocks its interaction with the ligand. Clinical studies have shown that anti‐PD‐1 has an acceptable safety profile, showing promising antitumor effects in melanoma, neuroendocrine tumors, uroepithelial carcinoma, and HCC, with significant economic advantages.^20^ However, using anti‐PD‐1 monotherapy alone is not enough to significantly improve the prognosis of patients with HCC. Multiple studies have reported the expansion of patient‐specific T‐cells after RFA, and this expansion has been shown to significantly correlate with the length of recurrence‐free survival (RFS) in HCC.^21^, ^22^, ^23^

Hence, we conducted this prospective study to evaluate the safety and efficacy of RFA combined with an anti‐PD‐1 treatment regimen by local plus systemic and physical plus immunotherapy for recurrent HCC.

## 2 PATIENTS AND METHODS

### 2.1 Study design

Our previous study showed a 1‐year RFS rate of 36.1% in patients with recurrent HCC who underwent local ablation. Assuming that the 1‐year RFS rate increases to 55.0% after combined anti‐PD‐1 therapy in patients with recurrent HCC, we calculated that 40 participants would be required, with 20 participants in each treatment arm, using a two‐sided type I error of 0.05 and a power of 80%.

The ethics committee has approved and registered this study with the Chinese Clinical Trials Registry (ChiCTR1900027807). This study is a prospective controlled trial, with RFS as the primary endpoint and safety as the secondary endpoint. Some patients choose to undergo regular check‐ups after RFA instead of opting for combined anti‐PD‐1 therapy due to financial constraints or personal reasons. As a result, this study is a non‐randomized concurrent controlled trial. Upon obtaining informed consent, the investigators divided the patients into a control group and an experimental group based on their individual preferences.

The inclusion criteria for this study are as follows: (1) Male or female participants aged 18 years or older. (2) Patients with a confirmed diagnosis of HCC who have experienced relapse within 6 months after RFA treatment or have undergone more than 2 RFA treatments. (3) Recurrent lesions that are suitable for RFA treatment. (4) The patient has one or more observable lesions in the liver, each ≤3 cm in size. (5) The patient has no extrahepatic metastases and no invasion of the portal vein or hepatic vein. (6) The patient has not received any other antitumor therapy, including radiotherapy, TACE, and targeted drugs, prior to this treatment. (7) Child‐Pugh grade A or B, with no current encephalopathy or ascites (small amount). (8) Left ventricular ejection fraction is ≥50%. (9) ECOG physical status is 0–1. (10) Baseline period laboratory tests (repeat laboratory tests are permitted during screening to assess subject eligibility, and before initiating the study treatment, the laboratory findings must conform to the protocol‐specific boundaries), namely, an absolute neutrophil count greater than 1.5 × 10^9^/L and a platelet count greater than 75 × 10^9^/L; Hemoglobin >10.0 g/dL (unless hemoglobin values have stabilized and the subject is in a stable cardiovascular state, asymptomatic and judged to be able to withstand the RFA operating procedure). Note: subjects might be given platelet or concentrated red blood cells transfusions based on clinical need and may undergo re‐evaluation once the condition has been addressed. Additionally, the baseline criteria for chemistry serum should be met, which include a serum creatinine level less than or equal to the upper limit of normal, a creatinine clearance of greater than or equal to 60 mol/min, a serum bilirubin level below 3.0 mg/dL, and a serum albumin level greater than 2.8 g/dL. (11) The patient voluntarily signs the informed consent form.

### 2.2 Patients

**FIGURE 1:**
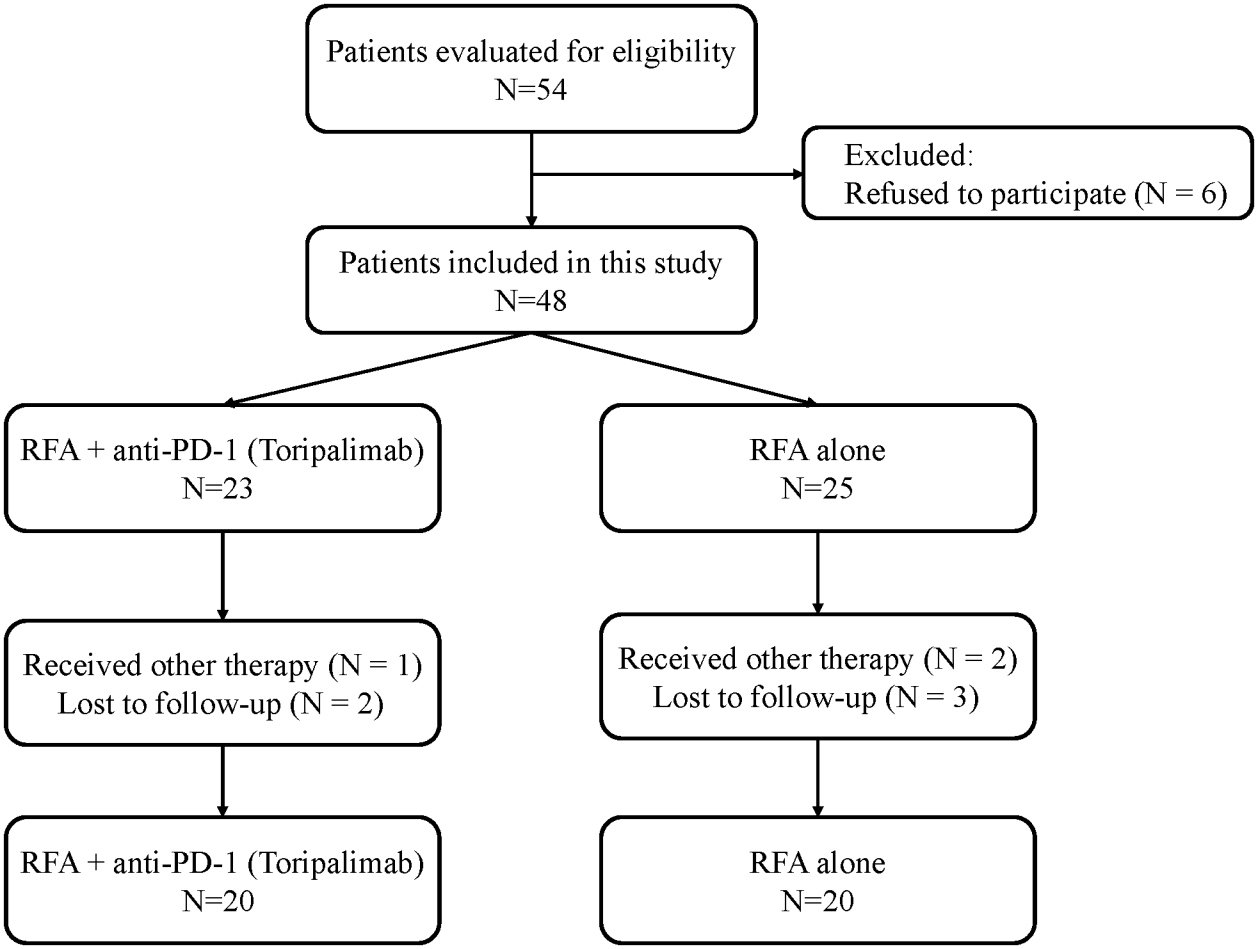
The flow chart of the subjects included in this study.

A flow chart of the subjects' inclusion is presented in Figure 1. Between September 24, 2019, and November 11, 2021, 54 patients with recurrent HCC were eligible for the study, and 6 patients refused to participate. Out of the 48 patients who remained, 23 were treated with a combination of RFA and anti‐PD‐1, while the remaining 25 received RFA alone. Throughout the follow‐up period, 8 patients were excluded either because they received other treatments or due to loss of follow‐up. The current study included a total of 40 patients (33 men and 7 women) with a mean age of 60 years. Among them, 20 patients received combination therapy (RFA combined with anti‐PD‐1 group), while the other 20 received RFA alone (RFA alone group). The follow‐up period concluded on April 10, 2022.

### 2.3 RFA therapy

The ablation procedure is performed using the RITA System. The choice of RFA needle type is based on the tumor size and morphology, which may include, but is not limited to, the Olympus RFA needle, etc. After routine disinfection, towel placement, and local anesthesia, a guide needle is inserted to puncture the liver parenchyma around the tumor in the liver. The puncture path and distance are determined, and a computed tomography (CT) scan is used to confirm the safe and accurate path. Once the CT scan confirms the safety of the ablation path and range, the ablation treatment begins. The number of ablation needles and duration of the procedure are determined by the investigator based on the specific condition. At the end of the procedure, the ablation puncture needle tract is routinely ablated, and the treatment needle is removed. Dressing is applied after the procedure. Repeat CT scans are performed to ensure that no complications such as pneumothorax have occurred.

The number of electrodes used for RFA procedures, whether it is a single electrode or multiple electrodes with overlapping ablations, is determined by the size of the tumor being treated. RFA is performed while the patient is under local anesthesia with intravenous analgesia or monitored anesthesia care. During the procedure, the output power starts at 50 W and is incrementally increased up to 200 W, with the power maintained until the impedance reaches its maximum value. To keep the temperature at the tip below 20°C, cold saline is injected into the electrode cavity using a pump. The RFA procedure is then carried out until the target lesion, identified by ultrasound or CT scan, including the entire tumor and border areas larger than 5 mm in size, has been treated. After completing the RFA procedure, an immediate contrast‐enhanced dynamic liver CT scan is performed, and a radiologist carefully reviews the results to assess technical success and identify any procedure‐related complications. In cases where the level of ablation is found to be insufficient, the RFA procedure is repeated during the same hospitalization.

### 2.4 Toripalimab management

Anti‐PD‐1 infusion (anti‐PD‐1 240 mg Q3W * 4 doses) will be administered within 7–10 days after RFA treatment and continued until the occurrence of an intolerable immune adverse reaction, completion of 4 doses, or tumor progression. After receiving the treatment, patients will return to the hospital for follow‐up at 1 month, 3 months, 6 months, and then every 3 months until the protocol‐specified treatment discontinuation event occurs. Subjects who discontinue treatment for reasons other than disease progression or death will also be monitored for tumor progression at the end of treatment.

### 2.5 Statistical analysis

Continuous variables that followed a normal distribution were presented using the mean and standard error, while non‐normally distributed continuous variables were presented using the median and quartiles. Student's *t*‐test was used to compare the correlations between treatment modality and baseline characteristics for continuous variables, and Fisher's exact test or *χ* ^2^ test was used for categorical variables. Survival curves were estimated using the Kaplan–Meier method, and differences were analyzed using log‐rank tests. Univariate and multivariate analyses based on Cox regression models were performed to identify independent prognostic factors associated with RFS. All statistical analyses were conducted using SPSS 24.0 software, and a significance level of *p* < 0.05 was considered statistically significant.

## 3 RESULTS

### 3.1 Baseline characteristics of all study patients

Nineteen patients (95.0%) started anti‐PD‐1 within 7–10 days after RFA. One patient started anti‐PD‐1 on the 14th day due to belly pain and fever. Nineteen patients (95.0%) completed 4 doses of anti‐PD‐1 and one patient (5.0%) discontinued treatment due to tumor progression.

The baseline characteristics of the 40 patients were summarized in Table 1. Almost all characteristics were evenly distributed between the two groups, including age, gender, Child‐Pugh score, albumin‐bilirubin index, etiology, HBV‐DNA index, alpha‐fetoprotein (AFP) level, number of RFA treatments, family history of HBV, smoking history, drinking history, platelet count, prothrombin time, number of tumors, and tumor size. There was no statistically significant difference between the two study groups in these aspects (*p* > 0.05).

**TABLE 1:** Baseline characteristics of all study patients.

	RFA+ anti‐PD‐1 (*n* = 20)	RFA (*n* = 20)	*p*‐Value
Age (mean ± standard deviation, years)	60 ± 6.612	59.9 ± 8.341	0.851
Gender			
Male	15 (75%)	18 (90%)	0.407
Female	5 (25%)	2 (10%)	
Child‐Pugh			
A	19 (95%)	15 (75%)	0.182
B	1 (5%)	5 (25%)	
ALBI			
1	3 (15%)	9 (45%)	0.082
2	17 (85%)	11 (55%)	
Etiology			
Hepatitis B	18 (90%)	19 (95%)	>0.9999
Hepatitis C	2 (10%)	1 (5%)	
HBV‐DNA index			
<1000 IU/mL	18 (90%)	20 (100%)	0.487
>1000 IU/mL	2 (10%)	0	
AFP			
<20 ng/mL	11 (55%)	15 (75%)	0.227
20–399 ng/mL	6 (30%)	5 (25%)	
≥400 ng/mL	3 (15%)	0	
Times of RFA			
≤2	9 (45%)	9 (45%)	>0.9999
>2	11 (55%)	11 (55%)	
Family history of HBV			
Yes	5 (25%)	9 (45%)	0.32
No	15 (75%)	11 (55%)	
Smoking history			
Yes	11 (55%)	6 (30%)	0.2
No	9 (45%)	14 (70%)	
Drinking history			
Yes	10 (50%)	8 (40%)	0.751
No	10 (50%)	12 (60%)	
Platelet count	127.4 ± 47.78	113.65 ± 14.39	0.448
Prothrombin time (s)			
≤13	17 (85%)	15 (75%)	0.695
>13	3 (15%)	5 (25%)	
Number of tumor			
≤2	17 (85%)	20 (100%)	0.231
>2	3 (15%)	0	
Size of tumor (cm)	1.35 (1.025, 2.475)	1.20 (0.85, 1.475)	0.161

Abbreviations: AFP, alpha‐fetoprotein; ALBI, albumin‐bilirubin; anti‐PD‐1, toripalimab; HBV, hepatitis B virus; RFA, radiofrequency ablation.

### 3.2 Efficacy of combination therapy

**FIGURE 2:**
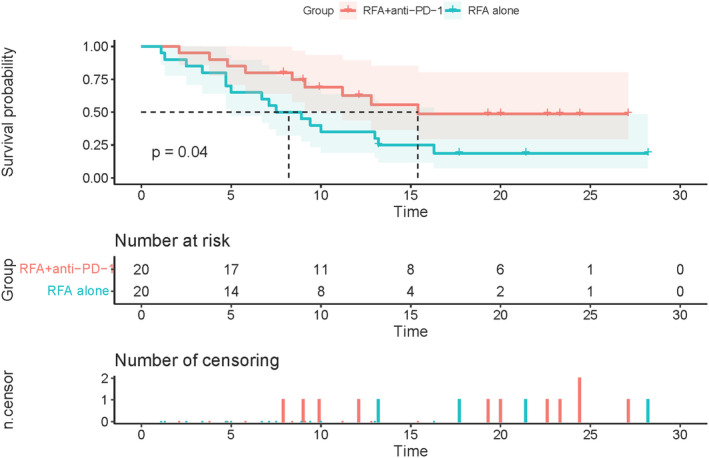
The RFS with combination therapy and RFA alone. RFA, radiofrequency ablation; RFS, recurrence‐free survival.

A total of 9 patients (45.0%) in the RFA combined with anti‐PD‐1 group and 16 patients (80.0%) in the RFA group showed tumor progression. As shown in Figure 2, 20 patients were given anti‐PD‐1 infusion after receiving RFA. The RFA combined with anti‐PD‐1 and RFA groups had a median follow‐up time of 22.6 and 21.4 months, respectively. The median RFS in the RFA combined with anti‐PD‐1 group was 15.40 months compared with 8.2 months in the RFA group (HR: 0.44, 95% CI: 0.198–0.961, *p* = 0.04). This suggests that the combined treatment modality significantly improved the RFS of patients (Figure 2). The combined RFA and anti‐PD‐1 groups demonstrated higher RFS rates compared to the RFA group alone at 6, 12, and 18 months, with rates of 80.0% versus 65.0%, 62.7% versus 35.0%, and 48.70% versus 18.8%, respectively. The difference between the two groups was statistically significant at 18 months (*p* = 0.04) (Table 2).

**TABLE 2:** The RFS at 6, 12, and 18 months in the RFA combined with anti‐PD‐1 and RFA groups.

	RFA+ anti‐PD‐1 (*n* = 20)	RFA (*n* = 20)	*p*‐Value
Median RFS (month)	15.4	8.2	0.0396
RFS rate (%)			
6 month	80%	65%	0.259
12 month	62.7%	35%	0.063
18 month	48.7%	18.8%	0.04

Abbreviations: anti‐PD‐1, toripalimab; RFA, radiofrequency ablation; RFS, recurrence‐free survival.

### 3.3 Changes in Child‐Pugh grade and AFP levels

After treatment, we evaluated the liver function reserve based on the Child‐Pugh score at baseline and the first follow‐up in both groups. The Child‐Pugh score situation was well balanced between the two groups of patients at baseline. As shown in Table 3, the results showed no significant changes in the liver function of patients at baseline and the first post‐treatment follow‐up in both groups (RFA combined with anti‐PD‐1 group, *p* > 0.999; RFA group, *p* = 0.107). One patient in the combined RFA and anti‐PD‐1 group experienced a decline in Child‐Pugh to grade B, while another patient in the same group showed an improvement in liver function to grade A. After combination therapy, the majority of patients maintained their liver function at the first follow‐up. Additionally, changes in AFP levels were assessed in both groups. Although there was no statistically significant difference, both groups showed a decreasing trend in AFP at the first follow‐up compared to baseline (*p* > 0.05; Table 3).

**TABLE 3:** Change of Child‐Pugh score and AFP for all study patients.

	RFA+ anti‐PD‐1 (*N* = 20)		*p*‐Value	RFA alone (*N* = 20)		*p‐*Value
	Baseline	First follow‐up		Baseline	First follow‐up	
Child‐Pugh						
A	19 (95)	19 (95)	>0.999	16 (75)	20 (100)	0.107
B	1 (5)	1 (5)		4 (25)	0	
AFP (ng/mL) (Q1, Q3)	7.750 (2.738, 147.725)	4.515 (2.080, 29.005)	0.738	4.005 (1.588, 22.803)	3.420 (1.745, 6.823)	0.565

Abbreviations: AFP, alpha‐fetoprotein; anti‐PD‐1, toripalimab; RFA, radiofrequency ablation.

### 3.4 Prognostic factors of RFS by COX regression

**FIGURE 3:**
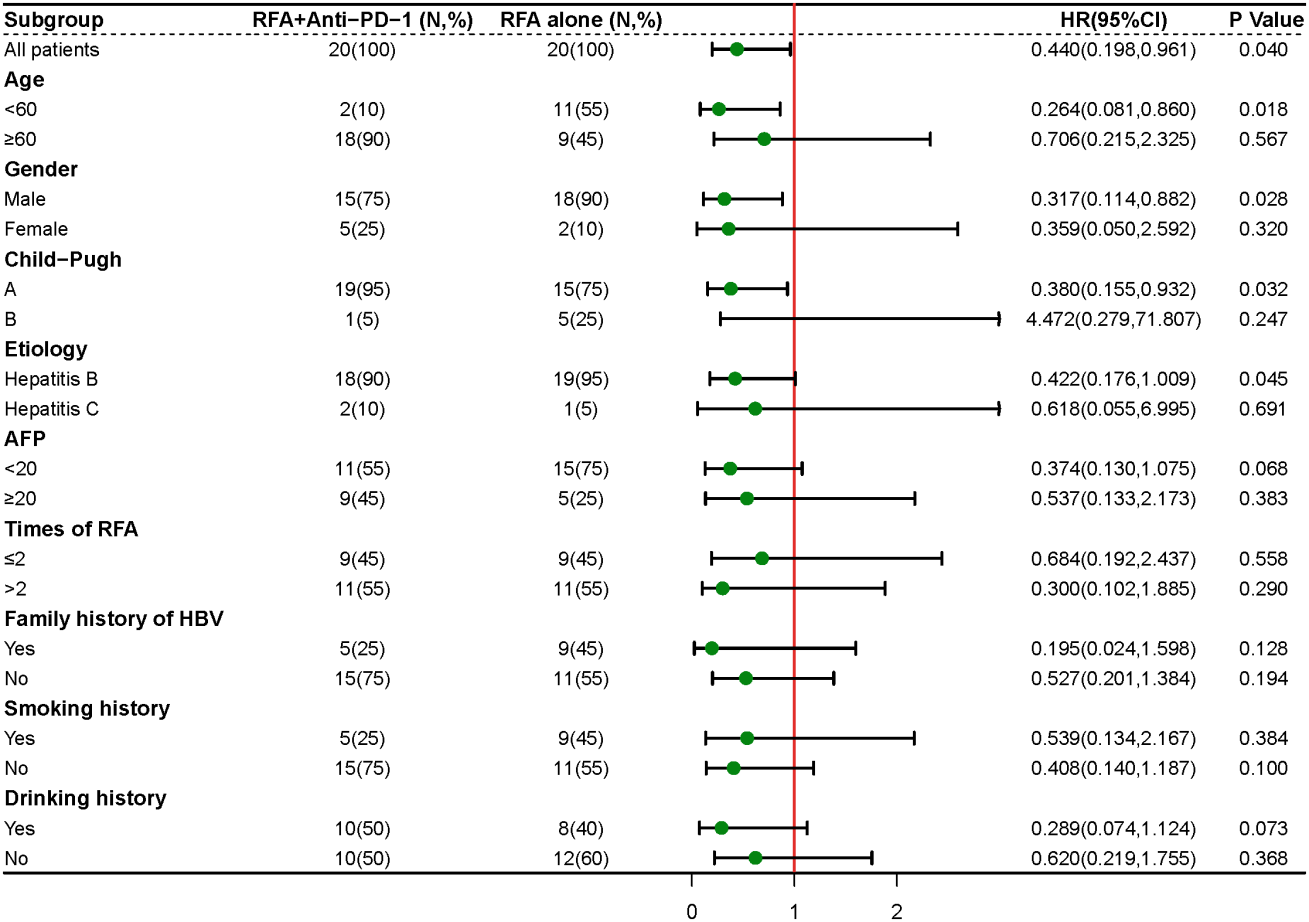
Subgroup analysis of RFS by Cox regression based on independent assessment. RFS, recurrence‐free survival.

We initially conducted COX regression analysis to further identify independent prognostic factors associated with patients' RFS. As shown in Table 4, the choice of treatment was identified as an independent prognostic factor for RFS in both the univariate and multifactorial Cox regression analyses. Moreover, based on the multifactorial analysis, the choice of treatment and gender were identified as independent risk factors for RFS. Subgroup analysis revealed that male patients younger than 60 years of age with a Child‐Pugh grade of A were more likely to benefit from RFA combined with anti‐PD‐1 therapy in terms of RFS (Figure 3).

**TABLE 4:** Prognostic factors of RFS by modified COX regression.

	Univariate analysis	Multivariate analysis	
	*p*‐Value	*p*‐Value	HR (95% CI)
Treatment choice	0.046	0.016	2.868 (1.220, 6.744)
Age (<60 vs. ≥60)	0.109	0.453	–
Gender (male vs. female)	0.068	0.016	3.372 (1.251, 9.088)
Child‐Pugh	0.490	–	–
AFP	0.916	–	–
Times of RFA (≤2 vs. >2)	0.743	–	–
Family history of HBV	0.610	–	–
Smoking history	0.133	0.464	–
Drinking history	0.305	–	–

Abbreviations: RFA, radiofrequency ablation; RFS, recurrence‐free survival.

### 3.5 Safety outcomes

The treatment of sudden adverse events (AEs) was primarily evaluated based on their frequency and severity grade, following the guidelines of the Common Terminology Criteria for Adverse Events version 4.03. Almost all patients experienced transient fever following RFA, which resolved within a short period of time in the majority of cases. As a result, we did not include these transient AEs caused by the procedure in our summary.

As shown in Table 5, the most common AEs of all grades in the RFA combined with anti‐PD‐1 and RFA alone groups were leukocyte decrease (45.0% vs. 70.0%), neutrophil decrease (20.0% vs. 55.0%), and platelet decrease (25.0% vs. 45.0%). In the RFA combined with anti‐PD‐1 group, immune‐related adverse events (irAEs) included rash (10.0%), skin pruritus (10.0%), and elevated thyroid hormone (5.0%). No statistical differences were observed between the two groups in terms of safety (*p* > 0.05). Therefore, the combination treatment was found to have an acceptable safety profile, with no unexpected safety signals identified.

**TABLE 5:** Treatment emergent adverse events.

Adverse events	RFA+ anti‐PD‐1 (*n* = 20)		RFA (*n* = 20)		*p*‐Value
	1–2 Grade	3–4 Grade	1–2 Grade	3–4 Grade	
Rash	2 (10%)	0	–	–	–
Skin pruritus	2 (10%)	0	–	–	–
Elevated thyroid hormone	1 (5%)	0	–	–	–
Diarrhea	0	0	–	–	–
Anemia	1 (5%)	0	2 (10%)	0	>0.9999
Neutrophils decreased	4 (20%)	0	7 (35%)	4 (20%)	0.5165
Leukocyte decreased	9 (45%)	0	10 (50%)	4 (20%)	0.1273
Platelet decreased	4 (20%)	1 (5%)	5 (25%)	4 (20%)	0.5804
ALT increase	2 (10%)	0	2 (10%)	0	>0.9999
AST increase	1 (5%)	0	1 (5%)	0	>0.9999
AKP decreased	1 (5%)	0	1 (5%)	0	>0.9999
Blood bilirubin increased	3 (15%)	0	5 (25%)	0	>0.9999
Prothrombin time prolonged	4 (20%)	0	5 (25%)	0	>0.9999
Hypoalbuminemia	4 (20%)	0	5 (25%)	0	>0.9999

Abbreviations: AKP, alkaline phosphatase; ALT, alanine aminotransferase; anti‐PD‐1, toripalimab; AST, aspartate aminotransferase; RFA, radiofrequency ablation.

## 4 DISCUSSION

This prospective study demonstrated that RFA combined with anti‐PD‐1 prolonged median RFS by 87.8% or 7.2 months compared to RFA alone in patients with recurrent cancer while showing the same effect in a subgroup analysis. In both univariate and multivariate analyses, the choice of treatment was identified as an independent risk factor for prognosis. Additionally, both the combination of RFA and anti‐PD‐1 and RFA alone showed an acceptable safety profile. Greten et al. reported a surprising response to topical therapy in combination with anti‐PD‐1,^24^ consistent with the results of our study. In conclusion, the research results suggest that in patients with recurrent HCC, RFA combined with anti‐PD‐1 may provide more clinical benefit compared to RFA alone.

Anti‐PD‐1 immunotherapy has exhibited good therapeutic effects in the treatment of advanced HCC. However, the efficiency of anti‐PD‐1 monotherapy is still unsatisfactory, and increasing the proportion of beneficiaries is currently a hot issue.^25^, ^26^ Chakraborty et al.^27^ reported that the activation of T cells was caused by the interaction between PD‐1 and PD‐1 ligands expressed by tumor cells. Therefore, restoring the function of effector CD8^+^ T cells was achieved by inhibiting PD‐1/PD‐L1. The activation of T cells induced by blocking PD‐1 could effectively lead to the killing of cancer cells. Most of the previous studies focused on combining anti‐PD‐1 with TACE or targeted drugs,^28^, ^29^ etc. In the present study, all patients underwent therapeutic RFA prior to anti‐PD‐1 therapy. RFA is a recommended radical treatment modality for early to mid‐stage liver cancer in the guidelines and has been shown to have satisfactory therapeutic effects in numerous previous studies.^30^, ^31^, ^32^ Previous studies indicate that RFA may enhance the antigenicity of tumors by decreasing tumor load, inducing immunosuppression in the body,^24^ exposing antigenic determinants on the surface of tumor cells, or altering tumor antigens.^21^ In situ inactivation through RFA produces heat shock proteins that can activate and induce a specific anti‐tumor immune response in the body.^33^ The necrotic tumor tissue after ablation, acting as allogeneic tissue, triggers the body's immune response and inhibits the secretion of tumor cytokines, thereby partially restoring the body's anti‐tumor immune response capability.^34^, ^35^ The above results suggest that RFA treatment can trigger an immune response and provide a better opportunity for PD‐1 blockade therapy.^36^ These theories were confirmed in our study, where RFA combined with anti‐PD‐1 prolonged the RFS of patients and was safely performed.

Furthermore, this study was conducted with a short‐term dosing period of only 3 months. Additionally, all patients completed four cycles of anti‐PD‐1 treatment. Studies have shown significant advances in immunotherapy for HCC,^37^, ^38^, ^39^ suggesting that after a certain duration of immunotherapy, positive results can be sustained for a longer period of time. This allows patients who benefit from immunotherapy to continue achieving stable results and experience long‐term survival. Immunotherapy can initiate or reset the “immune‐tumor” cycle in patients, amplifying the immune effects and providing long‐term immune protection for the body,^40^ This is crucial for eliminating residual tumor cells, preventing tumor recurrence and metastasis, and ultimately achieving long‐term survival.^40^ The results of our study demonstrated similar findings. At 6 and 12 months, there were no statistically significant differences observed in survival benefits between the two groups. However, at 18 months, the group that received RFA combined with anti‐PD‐1 treatment showed significantly better RFS compared to the group that only underwent RFA. Additionally, the RFS of the former group was superior to that of the latter group. Furthermore, the baseline information of the enrolled patients in our study was consistent, and we had better data integrity.

Our study similarly found that the combination of RFA and anti‐PD‐1 treatment was more effective in patients with advanced HCC. Among our enrolled patients, there was also a patient with stage C BCLC who chose to be treated with RFA combined with anti‐PD‐1 after relapse of previous treatment. This patient experienced significantly prolonged RFS, maintained grade A liver function, and did not experience any irAEs. The conclusion of our research aligns with previously reported findings, which have shown that anti‐PD‐1 alone or in combination with other therapies demonstrates promising antitumor effects in advanced HCC. Previously treated patients with advanced HCC showed an overall response rate (ORR) of 18.8% with anti‐PD‐1 monotherapy.^20^ The addition of RFA to anti‐PD‐1 treatment resulted in an increased response rate in these patients, without causing any treatment‐related deaths.^41^ A prospective phase II trial was conducted to explore the effect of the combination regimen, which showed an ORR of 63.9% and a median progression‐free survival of 10.5 months in 36 treatment‐naïve patients with advanced HCC.^42^

In our study, the results of the subgroup analysis showed that patients below the age of 60 benefited more from combination therapy compared to patients aged 60 and above. This difference in response may be related to alterations in the immune system, as suggested by Fane and Weeraratna.^43^ Demonstrated that immune aging leads to a decline in effector immune cells and subpopulations of overall immune function as the immune system becomes dysregulated with increasing age. The process is a result of a combination of factors, which include thymic atrophy, decreased numbers of naive T cells, impaired memory T cell function, and reduced diversity in T cell antigen recognition. As T cell function declines, natural killer cells, macrophages, and dendritic cells play a more prominent role in tumor recognition and suppression. Moreover, there is a phenotypic decline in cytotoxic activity that occurs with age. Liver function reserve not only affects the prognosis of patients but also influences the choice of treatment. Patients with Child‐Pugh A exhibit a better prognosis compared to those with Child‐Pugh B. More studies are needed to confirm whether gender affects the prognosis of patients.

In addition to its promising efficacy, RFA combined with anti‐PD‐1 has also proven to be safe. No treatment‐related deaths have been reported in this study. All grades of AEs associated with RFA in this study were leukocyte decrease, neutrophil decrease, and platelet decrease, which is consistent with previous findings.^44^, ^45^ There was no increase in the frequency of AEs related to RFA with the addition of anti‐PD‐1. Furthermore, the irAEs of any grade in the RFA combined with anti‐PD‐1 group included rash, pruritus, and elevated thyroid hormones, which is consistent with the findings reported by Zhang et al.^20^, ^46^, ^47^ all patients were successfully treated with standard management, and the slightly lower incidence of associated AEs in this study may be attributed to the short‐term use of anti‐PD‐1.

While discussing our findings, it is important to consider the limitations of our research. Firstly, the relatively small sample size of this study should be interpreted with caution. Our subgroup analysis found that male patients had a more favorable prognosis than female patients after receiving RFA combined with anti‐PD‐1 therapy, but this observation needs to be further confirmed in larger controlled trials. Secondly, the short follow‐up period did not allow for an assessment of overall survival, and further analysis is needed to evaluate the associated prognosis. Thirdly, this was a non‐randomized observational study, which introduces potential selection and ascertainment bias, despite efforts to balance baseline information. Additionally, the majority of patients enrolled in this study were infected with the hepatitis B virus, so further research is necessary to confirm these findings in other patient groups, including those with the hepatitis C virus.

In general, the findings indicate that combining RFA with anti‐PD‐1 therapy is a safe and effective treatment option for individuals experiencing recurrent HCC. It leads to a significant improvement in RFS and has an acceptable safety profile compared to using RFA alone. However, further investigations are necessary to determine the optimal timing and duration of RFA combined with anti‐PD‐1 therapy, as this remains controversial in reported clinical trials. Our results still require large sample, randomized, controlled clinical trials to validate and address relevant issues in the future.

## AUTHOR CONTRIBUTIONS

**Zhenyu Wen:** Data curation (equal); formal analysis (equal); methodology (equal); writing – original draft (lead). **Junxiao Wang:** Conceptualization (equal); supervision (equal); visualization (lead); writing – original draft (equal). **Bo Tu:** Conceptualization (equal); resources (equal); supervision (lead). **Yane Liu:** Data curation (equal); formal analysis (equal); software (equal); supervision (equal). **Yuqing Yang:** Investigation (equal); visualization (equal). **Li Hou:** Methodology (equal). **Xiang Yang:** Formal analysis (equal). **Xiaoyan Liu:** Funding acquisition (equal); writing – review and editing (lead). **Hui Xie:** Conceptualization (lead); funding acquisition (lead); methodology (lead); writing – review and editing (equal).

## FUNDING INFORMATION

This work was supported by grants from the Beijing Municipal Commission of Science and Technology (No. Z221100003522023).

## CONFLICT OF INTEREST STATEMENT

The authors have no conflict of interest.

### ETHICS STATEMENT

Approval of the research protocol by an Institutional Reviewer Board.

### CONSENT

Informed consent has been obtained from the patient.

### REGISTRY AND THE REGISTRATION NO. OF THE STUDY/TRIAL

Chinese clinical trial registry/ChiCTR1900027807.

### ANIMAL STUDIES

N/A.

## Data Availability

The data that support the findings of this study are available from the corresponding author upon reasonable request.
